# To be a professor: Academic mobility and publishing performance

**DOI:** 10.1371/journal.pone.0336133

**Published:** 2025-11-17

**Authors:** Martin Macháček, Aleš Melecký

**Affiliations:** Department of Economics, VSB-Technical University of Ostrava, Ostrava, Czech Republic; KTH Royal Institute of Technology, SWEDEN

## Abstract

Many aspiring academics target a professorship, but only some succeed. This study examines academic market concentration and its consequences in a post-communist country. We analyzed the field of economic sciences over a 22-year period, combining official data on the appointment process with manually collected data on applicants’ publishing performance in the Czech Republic. Using correlation and linear regression analyses, we investigated whether the mobility of candidates for full professorships is related to their research productivity and research visibility. Our findings revealed low migration flows among both domestic and international institutions. This resulted in high rates of inbreeding and potentially negative consequences, including a greater focus on local journals and lower publication performance after appointment. On average, internal candidates produce more publications, but fewer of them are written in a foreign language. This renders them virtually non-existent to the international research community. After becoming professors, internal candidates are also more likely to reduce their publication performance. Implementing a national, performance-based research funding system has yielded favorable results, such as increased publication performance among candidates over time. These effects were significantly higher in the capital, driven by the top Czech university (Charles University). Despite significant policy reforms, Czech higher education still suffers from considerable path dependence. Government bodies should promote competition among higher education institutions through regulation and financing. We discuss several measures that would modify the existing competence promotion model and support the international competitiveness of the higher education sector.

## 1. Introduction

Since the collapse of communism in the early 1990s, the former socialist countries of Central Europe (CE) have successfully transformed their political systems and joined the OECD, EU and other ‘rich country clubs.’ Despite tremendous efforts to transform their ‘previously ideologically driven, overregulated, inefficient’ higher education (HE) systems into ‘innovative conveyers of human capital for the 21^st^ century economy’ [1, p. 523], these countries have not become part of the premium segment of the global system of knowledge production [2,3]. Their HE systems still lag behind those of Western Europe [4]. Higher education institutions (HEIs) in the CE region typically occupy low positions in various performance rankings, especially those related to research performance, prestige, and reputation [2]. Despite the fall of the Iron Curtain and the removal of formal barriers to academic mobility, internationalization remains low in these countries [5]. This limits the inflow of fresh ideas and may contribute to technological and economic stagnation and the rise of populism [6].

Structural characteristics of HEIs in CE countries can limit their performance in the global HE market. These characteristics include persistent underfunding and related academic brain drain [1,2]. One of the main challenges is the persistence of a rigid academic environment that does not sufficiently motivate staff mobility [7] and does not curb rampant academic inbreeding [8–12]. Low mobility of academics reduces their connection to external research networks, which may make them less productive or influential [13–15]. Consequently, national knowledge production and human capital accumulation will suffer.

In this paper, we examine the production of full professors in the Czech Republic, who represent the primary segment of the academic labor market and have the greatest impact on knowledge production. Specifically, we investigate whether the mobility of candidates for full professorships in economics is related to their research productivity and research visibility. The country’s decentralized promotion system, which includes internal and external candidates for professorships, allows for a comparative performance analysis. We address the following three research questions: Do internal candidates publish more or less than external candidates? Do internal candidates publish more or less visible articles than external candidates? Are professors who were home-grown more prone to a decline in productivity after achieving professorship than those who went through the appointment process at another institution? We use the number of publications in the Web of Science (WoS) database and their language as proxies for academic merit, visibility, and impact to address these questions.

The Czech HE system can be characterized as dual and highly heterogeneous. It includes various non-university HEIs focused on education and human capital formation at the bachelor’s degree level (and sometimes even the master’s degree level), as well as PhD-awarding universities. However, only some of the PhD-awarding universities have the right to promote candidates to the rank of associate professor (‘docent’) and even fewer can promote them to the rank of (full) professor. The National Accreditation Bureau for Higher Education grants these rights to HEIs for a limited period based on a thorough analysis of their research performance and human resources [16]. Authorized universities may promote their own academic staff, as well as applicants from other HEIs and non-HEIs, including research institutes, government departments, and businesses. There are no unified national rules for the professor appointment procedure, and each university sets out the details in its internal regulations. Therefore, the specific qualification requirements on applicants vary across universities. Nevertheless, the appointment process always includes a review of the applicant’s pedagogical and scholarly credentials, including an evaluation of their research, teaching, and other professional accomplishments. Furthermore, the Scientific Board – chaired by the rector (the administrative and ceremonial leader of a Czech university, roughly equivalent to President at American universities) and composed of at least one-third external members – votes by ballot on whether to nominate the candidate for professor. After a professorship is awarded, it is nationally recognized and easily transferable to other HEIs or non-HEIs. It is also valid for a lifetime. In other words, in the Czech Republic, professor denotes both professional position and degree.

In theory, a Czech university can fill a junior academic position with a professor, but this rarely happens in practice. Unlike in Italy, France, Belgium, Spain, Germany, or Slovenia, professors in the Czech Republic are not civil servants, but rather university employees who typically have permanent contracts. Professors have traditionally enjoyed a high social status. Their degrees are automatically recognized in countries that have signed intergovernmental agreements with the Czech Republic, such as Poland (since 2006) and Slovakia from 2001 to 2015, as well as in countries whose Ministry of Education has granted recognition, such as Slovakia since 2015. Therefore, a professorship is clearly attractive to both individual applicants and HEIs, as government regulations require all accredited degree programs to be guaranteed by qualified academic staff (see [16] for more details). Unlike the chair system in Germany and Austria, several associate and full professors typically work together in a department, which may be headed by an assistant professor or higher.

Currently, all applicants for professorships, as well as appointed professors, are expected to publish in WoS journals listed in their respective fields. This reflects institutional reforms of the past two decades that have transformed Czech academia from a teaching-oriented community into a research-intensive, internationally competitive system of knowledge production. Since 2004, the Methodology for Evaluating Results of Research Organisations and Completed Programs has been in force and has served as a periodic evaluation of nationwide research performance [17,18]. Since 2010, the results of these evaluations have been used to allocate public funding to research organizations. Consequently, HEIs have become both educational institutions and research organizations, legally and financially. Therefore, their current and future professors are urged to follow the standard ‘three-mission model’ of teaching, research, and community engagement.

We hypothesize that the limited, administratively regulated supply of professorships, coupled with substantial individual and institutional revenues from professorships, creates perverse incentives for candidates, appointed professors, and universities. This is consistent with the findings of OECD experts nearly twenty years ago [19]. As the supply of professors remains limited, we expect less pressure on their academic mobility and research performance both before and after their appointment. More specifically, we expect most candidates to be internal with a lower number of publications in less visible outlets. We also expect these internal candidates to be more likely to experience a decline in productivity after being appointed, thus becoming “academic deadwood” [20]. Furthermore, our research builds on the extensive literature regarding in-group bias, favoritism, and social ties in academia. This stems from the idea that the composition of scientific boards and candidate gender may play a role in academic promotion [21–26].

Since the research productivity and visibility of academics may be influenced by factors other than their internal or external status and gender, we also consider the prestige of the university where the appointment process took place, as well as spatial and time-related factors. We acknowledge that the quality of scientific contributions is often more significant than the quantity, particularly when evaluating international collaborations or academic visibility. Nevertheless, we contend that employing the number of publications as a metric for academic performance, without considering journal quality or citation impact, is a valid approach when the country’s publication output predominantly appears in local Czech- and Slovak-language journals with modest impact factors and article influence scores [27,28]. This is supported by the fact that, from 2010 to 2018, the Czech Republic operated a research assessment and funding system that prioritized publication quantity over quality [17]. Additionally, Sandström and van den Besselaar [29] found a strong positive correlation between the number of papers and the number of citations. This correlation also held true for the production of high-impact papers and regardless of gender [30].

As expected, most professorship candidates in the Czech Republic (78%) were internal, indicating low mobility in this prominent academic staff segment. Differences in qualification requirements for professors across universities may motivate some candidates to apply for a professorship at another institution, either to find less rigorous conditions or greater prestige. However, most candidates clearly do not take advantage of this opportunity. There may be several reasons for this, including the existence of closer ties between internal candidates and rector-led scientific boards. We found that contrary to our expectations, the publication performance of internal candidates is not significantly different from that of external candidates, but visibility of their research may differ. These candidates produce more publications on average; yet, fewer are written in a language other than Czech, rendering them virtually non-existent to the international research community. This finding is consistent with existing work on the less visible and influential research of immobile academics [13,14]. Moreover, as expected, internal candidates are more likely to reduce their publication performance once they become professors.

Although our results are based on the analysis of only one discipline, policy makers should still consider them. Communist ideology greatly affected the social sciences and humanities (SSH) [31], which struggled with low prestige and limited international scientific connections. Conversely, modern economics is a discipline that significantly impacts social development [32] and is dominated by core theories and methods, with shared standards of excellence [33]. It produces knowledge that can be transferred globally, greatly facilitating the mobility of scholars [34]. Therefore, the profound post-communist transformation of the knowledge production process should be clearly visible here. If elite academics remain immobile and continue to produce little internationally recognized output, policymakers should consider fundamental reforms to the entire HE system. Furthermore, evidence suggests that similar issues exist in other disciplines [35]. Thus, our paper discusses possible policy measures that could improve the situation.

The rest of this paper is organized as follows: Section 2 presents the related literature. Section 3 describes the employed data and empirical methodology. Section 4 discusses the empirical results, including migration patterns and publication trends. Section 5 concludes the argument.

## 2. Related literature

Compared to developed Western countries, both domestic and international academic mobility remain low in post-communist countries. In the Czech academic environment, Macháček and Srholec [36] reported that 67% of social scientists affiliated with Charles University started their research careers at this institution, while 25% came from other Czech HEIs, and only 15% started their careers abroad. The authors found similar results for HEIs in other CE countries. More recently, Macháček et al. [5] found that the Leiden Ranking institutions in Central, Eastern, and Southern Europe had the highest proportion of insiders and observed no disciplinary differences. Fischer and Lipovská [10] showed that the broader inbreeding ratio calculated for economics associate professors in the Czech Republic was 49%, but with significant heterogeneity across universities.

At the same time, CE countries typically have comparatively low research productivity and visibility in leading scientific journals, especially in the academic disciplines covered by SSH, including economics [37]. For example, Jurajda et al. [35] analyzed the WoS publication performance of six post-communist EU countries, including the Czech Republic, relative to six comparably sized EU-15 countries. They found that the CE countries still lag significantly behind their Western counterparts in the social and medical sciences. Additionally, Macháček and Srholec [27] found that about one-fifth of the publications indexed in the Scopus citation database in the Czech Republic are concentrated in Czech journals and mostly produced by domestic authors. Similarly, Münich and Škoda [28] showed that Czech publication output is typically concentrated in Czech and Slovak journals, with relatively low impact factors and article influence scores.

Literature on research performance suggests that there is a correlation between academic mobility and publication productivity and visibility [13,14]. Mobility relates to the extent and structure of researchers’ professional networks, reflected in externalities and spillovers of knowledge and skills, division of labor, specialization, and innovation in research. Regarding international mobility, most literature indicates a positive relationship with research performance [38–43], though the results are not entirely conclusive [44]. Given this, the low international mobility of academics working at Czech universities (including full professors) is clearly an important source of their lack of research productivity and visibility.

A positive influence of inter-university mobility to “better” departments (i.e., upward mobility) on research performance and vice versa can be identified mainly in the Anglo-Saxon academic system [45]. Meanwhile, a rich discussion of the peculiarities of the European and American economic and academic markets points to differences in size, structure, openness, competition, and related research and mobility strategies [46–51]. Empirical evidence from Norway [52] and Sweden [53] has shown that mobility has no significant effect on bibliometrically measured research performance, at least in SSH disciplines. Similarly, Abramo et al. [54] analyze Italy’s public, highly centralized academic system with little autonomy at the university level, and show that national mobility is not positively correlated with research productivity. They also demonstrate that over half of the academics performed worse after transferring to other HEIs, consistent with literature indicating significant productivity decreases in the years following job changes due to “adjustment costs”.

In the Czech Republic, the government strictly regulates the domestic academic sector, yet universities have the right to award nationally recognized professorships. This raises the question of whether the inter-university mobility of candidates for economics professorships correlates with their publication record and the international visibility of their publications. If internal candidates typically have fewer journal articles or fewer articles published in journals accessible to the international scientific community, this will indirectly suggest possible in-group bias and favoritism within the decision-making bodies, i.e., university scientific councils. This is because economics is presented as a profession with a well-defined job hierarchy [55], where the number of publications, citations, and research grants plays a key role in academic promotion at all types of universities [56].

Existing literature suggests that even after establishing a merit-based peer review process, in-group bias based on discriminatory practices, such as favoritism and nepotism, may still play a role [57]. This applies to journal publication [58–65], grant awards [66], selection of members of learned societies [67–69], and hiring and promotion [22,23,70,71]. While some authors have found editorial favoritism to have a positive impact on the quality of published journal articles due to existing information asymmetry [72] and better information for editors [59,60], selection based on acquaintances and personal ties in the academy is generally considered detrimental. For instance, Fisman et al. [67] found that hometown ties increased the probability of selection for fellowships in the Chinese Academies of Sciences and Engineering by 39%. Additionally, selected candidates with hometown ties were half as likely as selected fellows without ties to have a high-impact publication. Privileged members of selection committees often become biased, losing their objectivity and independence. This bias can stem from fear or anticipation of retribution, creating an inherent moral hazard in the selection process. In the Czech Republic, university research councils often include deans, vice-deans, and other members of individual faculties (schools) who nominate candidates for professorships. This can result in implicit cooperation and support among faculties for internal candidates, which undermines objective and independent peer review.

The tenure and promotion literature has long discussed the existence of “academic deadwood”, which occurs when the award of a professorship leads to a subsequent decline in the research productivity of the candidate [20]. However, empirical results are mixed. Some studies have found no negative impact of promotion on the number of research publications and grants [73,74]. However, Brogaard et al. found that, in a sample of all academics in the top 50 U.S. economics and finance departments from 1996 to 2014, both the average number of publications and the average number of “home runs” (i.e., highly influential publications) declined by about 30% annually in the two years following tenure [75]. The phenomenon of “academic deadwood” is commonly explained by loss of research motivation [76] and fear of sanctions for inadequate research [77] after obtaining a professorship under the promotion model. The age of the researcher and the life-cycle nature of research productivity are also important factors [78–83]. According to the well-known principle of the cumulative advantage (also known as the Matthew effect, e.g., [84–86]), professors who were appointed at a young age may not experience a decline in productivity after achieving full professorship, as documented by Abramo et al. [87] for Italy. These professors may even increase their research productivity.

In the Czech Republic, the average age of appointed professors has been decreasing quite rapidly. It was 63 years in 1991, 54 years in 2001, and only 51 years in 2012 [88,89]. This trend suggests that the phenomenon of “academic deadwood” related to age has diminished over time. Because of the hypothesized in-group bias of the scientific council members, one might expect professors appointed internally will be more prone to loss of research productivity compared to professors appointed at other universities. This is consistent with the findings of Zinovyvea and Bagues [23], who showed that full professors in Spain with ties to members of their promotion committee published less after promotion. Similarly, Bian et al. [71] documented the detrimental effect of relationship hiring on candidates’ future publication performance using a sample of German professors.

The motivation to conduct and publish research is also influenced by the national system of research evaluation and funding and its changes. Traditionally, full professors have been expected to conduct research and publish their findings. However, until recently, a significant number of economics professors in both the Czech Republic and Slovakia were appointed without having authored a single WoS publication [90,91]. This aligns with the role of universities under communism, when their primary focus was teaching and producing manpower for the needs of the centrally planned economy. Research was the responsibility of the Institutes of the Academy of Sciences [92].

In 2010 the Czech Republic launched its national performance-based research funding system (PRFS). Over the past 25 years, many countries have introduced such systems to distribute public funds among HEIs based on their research output, particularly using bibliometric indicators. The Czech system is based on a “citation-based model”, as are systems in Poland and Slovakia [93]. A similar mechanism has subsequently been adopted by many HEIs. International studies have found that introducing the PRFS influences researchers’ publication behavior, especially in the fields of social sciences and humanities, including economics [94–100]. To promote the international competitiveness of their research, academics should strategically alter their publication behavior. This has been partially confirmed [101–103].

Several studies identified changes in Czech publication patterns, suggesting the potential impact of the national evaluation and funding system [103–105]. Vanecek and Pecha [103] found that the annual local production of WoS publications increased by over threefold between 2000 and 2015. However, the number of articles increased only 2.6 times, while the number of conference proceedings papers increased eightfold. The fastest growth in the number of proceedings papers occurred in the 17 social science fields, and this trend began after the introduction of the national PRFS. Regarding economics, Petr et al. [104] showed that the number of articles registered in WoS increased by 47% and number of articles in Q1 and Q2 journals by 26.7% in the Czech Republic in 2016. Macháček et al. [106] examined the publication performance of full professors of economics appointed since 1999 and found an increasing trend in WoS-indexed publications. However, this trend was driven by lower-ranked local journals and conference proceedings rather than high-quality international journals. This could be seen as a natural consequence of strategies employed by academics to secure promotion by publishing numerous mediocre papers rather than a few excellent ones [107]. In a recent related study on the effects of Ukrainian policy reform, Abramo et al. [108] confirmed that introducing Scopus and WoS publication requirements as an incentive for professorships worked. However, in several cases the increase in research output came at the expense of research impact.

Several individual- and institutional-level factors influence the research productivity and visibility of academics. These factors include gender, the prestige of the institution with which the researcher is affiliated or where they seek a professorship, and regional disparities in resource distribution. [109,110]. For instance, numerous studies have identified and attempted to explain gender differences in productivity [e.g., 108,109] and the existence of gender gaps in promotion [24–26,111–115]. Among organizational factors, university prestige and budget have been identified as important determinants of publication performance and research impact [116–118]. Additionally, the regional environment in which universities operate may also have a significant impact. Variables such as regional GDP per capita, gross expenditure on research and development, and the share of tertiary education are positively related to research productivity [119,120]. Therefore, we include gender, university prestige, and the regional environment as explanatory variables in the regression models to reduce the risk of oversimplified conclusions.

## 3. Data and methodology

### 3.1. Data

Our unique dataset combines information from two sources. First, it includes a publicly available database of professorships from the Ministry of Education, Youth, and Sports. The Ministry publishes information on the initiation and results of habilitation and professor appointment procedures in this database. It is accessible via the following link: https://www.msmt.cz/vzdelavani/vysoke-skolstvi/rizeni-ke-jmenovani-profesorem?lang=1/. This is specified in Section 75(3) of Act No. 111/1998 Coll., on Higher Education Institutions and on Amendments and Supplements to Other Acts (“the Higher Education Act”). Basic information about the Ministry’s processing of personal data can be found here: https://msmt.gov.cz/contacts-and-informations/basic-information-about-personal-data-processing-by-the. Second, the dataset includes individual data from the WoS on applicants’ publication performance, specifically the number of articles published in WoS-indexed journals, which were manually selected. If deemed necessary, we double-checked the authorship of publications via personal webpages or institutional systems. It is worth noting that names are less likely to be confused in the Czech language because the gender is usually clearly identifiable in the name. In Czech, as in some other Slavic languages, grammar rules require different suffixes for masculine and feminine versions of the same surname for a large majority of “native” surnames. This means that, in most cases, surnames of male and female family members do not exactly match. Additionally, there are not enough researchers in the country for name duplication to be common. Most universities also provide information about staff publications, including as part of personal profiles on university websites. Finally, the number of professors in the field of economics, as defined above, is still relatively small. Overall, the risk of confusing profiles is negligible.

The data were collected between October 2020 and March 2021. To reduce potential mistakes, we applied the four-eyes principle; that is, the two authors examined the data independently. We confirm that the collection and analysis methods complied with the terms and conditions of the data source. The dataset supporting the findings of this study is available in Excel file S5 Dataset within the supplementary materials. Our dataset contains records of 216 appointment procedures and covers a 22-year period from 1999 to 2020. Regarding the distribution of sex, most applicants were male (160) and about a quarter (56) were female. Most applicants were in-house (169), whereas the rest came from other institutions (47), mostly from the Czech Republic and Slovakia.

### 3.2. Methodology

#### Correlation and linear regression analysis.

To further exploit our data on individual publication performance, we used pairwise correlation analysis, which measures the strength of a linear relationship between two variables, to create a correlation matrix for the variables of interest. The correlation coefficient *r* is calculated as follows:


$$r=\frac{n\sum xy-\sum x\sum y}{\sqrt{\left(n\sum x^{\text{2}}-{\left(\sum x\right)}^{\text{2}}\right)\left(n\sum y^{\text{2}}-{\left(\sum y\right)}^{\text{2}}\right)}}$$ (1)

Where *x* and *y* are values of variables, and *n* is sample size.

In the next step, we conducted a regression analysis to estimate the effects of the internal applicant dummy variable (1 = internal applicant, 0 otherwise), the gender dummy variable (0 = male, 1 = female), and the year of professorship (Year) on the number of published articles (NOA_T), which served as a proxy for research productivity (e. g., [91,121]). In addition, we consider the number of articles published in English and in a few cases German and Russian, especially in older publications (thereafter for simplicity referred as articles in English, ENG_NOA_T) as a dependent variable, which serves as a proxy for research visibility and impact, as the paper has a greater international reach due to lower language barriers [122]. Although we do not consider traditional or alternative citation metrics, we believe that articles published in English are typically cited more frequently by the international community due to their language accessibility and more frequent representation in WoS-indexed journals [123–125]. This is one reason why journals published in countries where English is not the native language are switching to or launching in English [126]. ε_*i*_ represents the idiosyncratic disturbance term (residual). In robustness tests, we extend the baseline models by adding dummy variables to examine the effects of institutional quality, access to funding, and regional disparities. First, we test the effect of location and structural characteristics of regions (Equations 4 and 5). We create a dummy variable D_Capital for Prague, which is the primate city of the Czech Republic and attracts academics. Three universities with major economics departments are located in Prague, namely ČZU, VŠE, and UK. In addition, we include D_Structural to control for structurally affected regions in the periphery that suffer from brain drain. In the Czech Republic, the government legally identifies some of the country’s 14 administrative regions as “structurally affected” for the purposes of additional development aid. Currently, this status applies to the Ústí, Karlovy Vary, and Moravian-Silesian Regions. The only structurally affected region that actively provides professorships in the examined field is the Moravian-Silesian region, in which two universities of interest are located – OSU and VŠB-TUO. Second, we introduce dummy variables to control for top universities in the examined field, which are motivated by university rankings and reflect their higher funding from both the state budget and project funds (Equations 6–7). We introduce dummy variables for two A-ranked universities, namely D_UK for Charles University in Prague and D_MU for Masaryk University in Brno. The dummy variables take the value 1 if the given professorship was held at these institutions and 0 otherwise.


$${NOA\_T}_{i}=\;\beta_{\text{0}}+\;{\beta_{\text{1}}IN}_{i}+\;{\beta_{\text{2}}SEX}_{i}+{\beta_{\text{3}}Year}_{i}+\varepsilon_{i\;}$$ (2)

$$ENG\_{NOA\_T}_{i}=\;\beta_{\text{0}}+\;{\beta_{\text{1}}IN}_{i}+\;{\beta_{\text{2}}SEX}_{i}+{\beta_{\text{3}}Year}_{i}+\varepsilon_{i\;}$$ (3)

$${NOA\_T}_{i}=\;\beta_{\text{0}}+\;{\beta_{\text{1}}IN}_{i}+\;{\beta_{\text{2}}SEX}_{i}+{\beta_{\text{3}}Year}_{i}+\text{D}\_{\text{Capital}}_{i}{+\text{ D}{\_\text{Structural}}_{\text{i}}+\varepsilon }_{i\;}$$ (4)

$$ENG\_{NOA\_T}_{i}=\;\beta_{\text{0}}+\;{\beta_{\text{1}}IN}_{i}+\;{\beta_{\text{2}}SEX}_{i}+{\beta_{\text{3}}Year}_{i}+\text{D}\_{\text{Capital}}_{i}{+\text{ D}{\_\text{Structural}}_{\text{i}}+\varepsilon }_{i\;}$$ (5)

$${NOA\_T}_{i}=\;\beta_{\text{0}}+\;{\beta_{\text{1}}IN}_{i}+\;{\beta_{\text{2}}SEX}_{i}+{\beta_{\text{3}}Year}_{i}+{\text{D}\_\text{UK}}_{i}{+\;D{\_MU}_{i}+\varepsilon }_{i\;}$$ (6)

$$ENG\_{NOA\_T}_{i}=\;\beta_{\text{0}}+\;{\beta_{\text{1}}IN}_{i}+\;{\beta_{\text{2}}SEX}_{i}+{\beta_{\text{3}}Year}_{i}+{\text{D}\_\text{UK}}_{i}{+\;D{\_MU}_{i}+\varepsilon }_{i\;}$$ (7)

## 4. Results and discussion

### 4.1. Place of appointment

**Fig 1:**
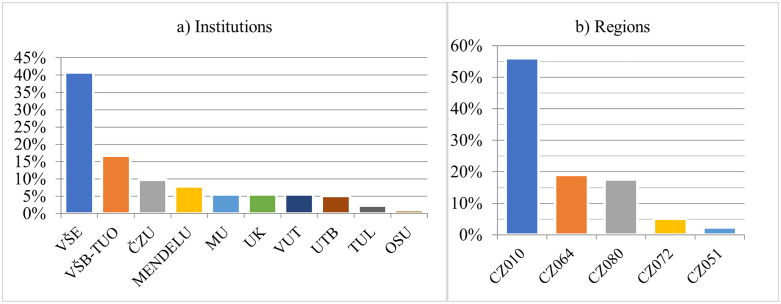
Place of appointment. *Note:* See S1 Table for a list of institutions and their abbreviations. CZ010 – Prague, CZ064 – South Moravian Region, CZ080 – Moravian-Silesian Region, CZ072 – Zlín Region, CZ051 – Liberec Region.

As mentioned above, the number of HEIs allowed to promote candidates to the rank of professor is limited. In the Czech Republic, only 10 public institutions conducted appointment procedures. Fig 1(a) illustrates their market shares during the 1999–2020 period.

More than 40% of the appointment procedures were conducted at a single HEI (VŠE). The dominant position of this university is not surprising given its long tradition, specialization in economics and business, and location in the capital. As Fig 1(a) shows, the relevant academic market in the Czech Republic is quite concentrated, with three institutions (VŠE, based in Prague; VŠB-TUO, based in Ostrava; and ČZU, also in Prague) accounting for two-thirds of the appointment procedures. Therefore, it may be desirable to increase competition among institutions in the future.

Fig 1(b) illustrates the geographical distribution of appointment procedures in the five regions. From a spatial perspective, there is a high concentration in the relevant market, with 56% of appointment procedures conducted in the Prague region, where three universities are located (VŠE, ČZU, UK), followed by the South Moravian region (19%; MENDELU, MU, VUT) and the Moravian-Silesian region (17.6%; VŠB-TUO, OSU). From this perspective, the regions of Zlín (5.1%) and Liberec (2.3%) had only a small share. No appointments were made in the remaining nine regions of the Czech Republic. The Prague region forms the core of the market, followed by clusters in the South Moravian and Moravian-Silesian regions.

### 4.2. Migration of applicants across institutions

**Fig 2:**
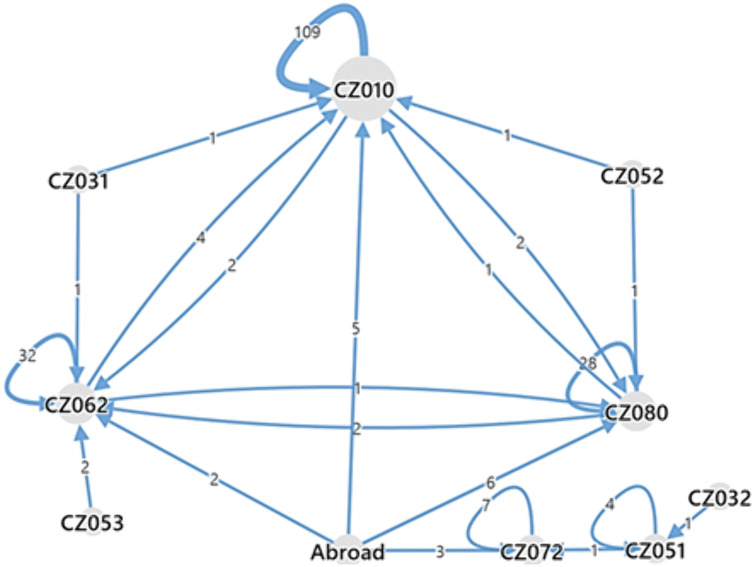
Migration of applicants across regions. *Note:* CZ010 = Prague, CZ031 = South Bohemian Region, CZ032 = Plzeň Region, CZ051 = Liberec Region, CZ052 = Hradec Králové Region, CZ053 = Pardubice Region, CZ062 (CZ064) = South Moravian Region, CZ072 = Zlín Region, CZ080 = Moravian-Silesian Region.

**Fig 3:**
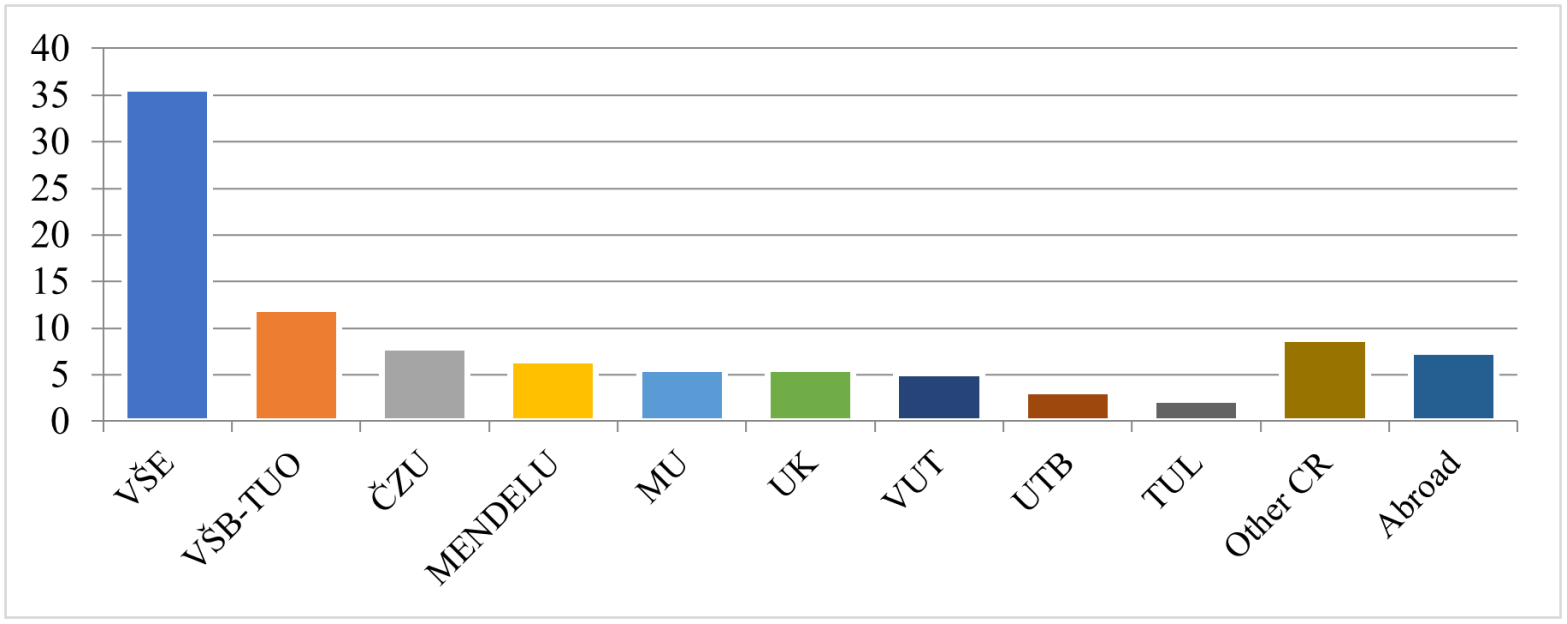
Home institution of applicants. *Note:* See S1 Table for the list of institutions and their abbreviations.

To analyze the flows across institutions, Fig 2 shows the migration flows from an applicant’s home institution to the institution where the appointment procedure took place, and Fig 3 shows the distribution of applicants’ home institutions.

As confirmed by the migration map in Fig 2, the core of the appointment procedures was located in the Prague region. However, this region has only limited connectivity to the other regions that are not directly connected to the Prague region (Plzeň, Liberec, Pardubice, and Zlín), although three of them are geographically close. The other two clusters are the South Moravian and the Moravian-Silesian regions, both of which are well connected to domestic and foreign institutions through appointment procedures.

Fig 3 illustrates the demand for professorships with respect to applicant home institution. With more than 36% of all applicants, VŠE has the highest demand for appointments in the Czech Republic. The three institutions with the highest shares (VŠE, VŠB-TUO, and ČZU) account for more than 55% of applicants. Interestingly, women are more likely to migrate than men. Based on the relative share of migrants by gender (e.g., out_women/women*100), we find that 30% of women started their professorship at another institution, compared to only 19% of men.

**Fig 4:**
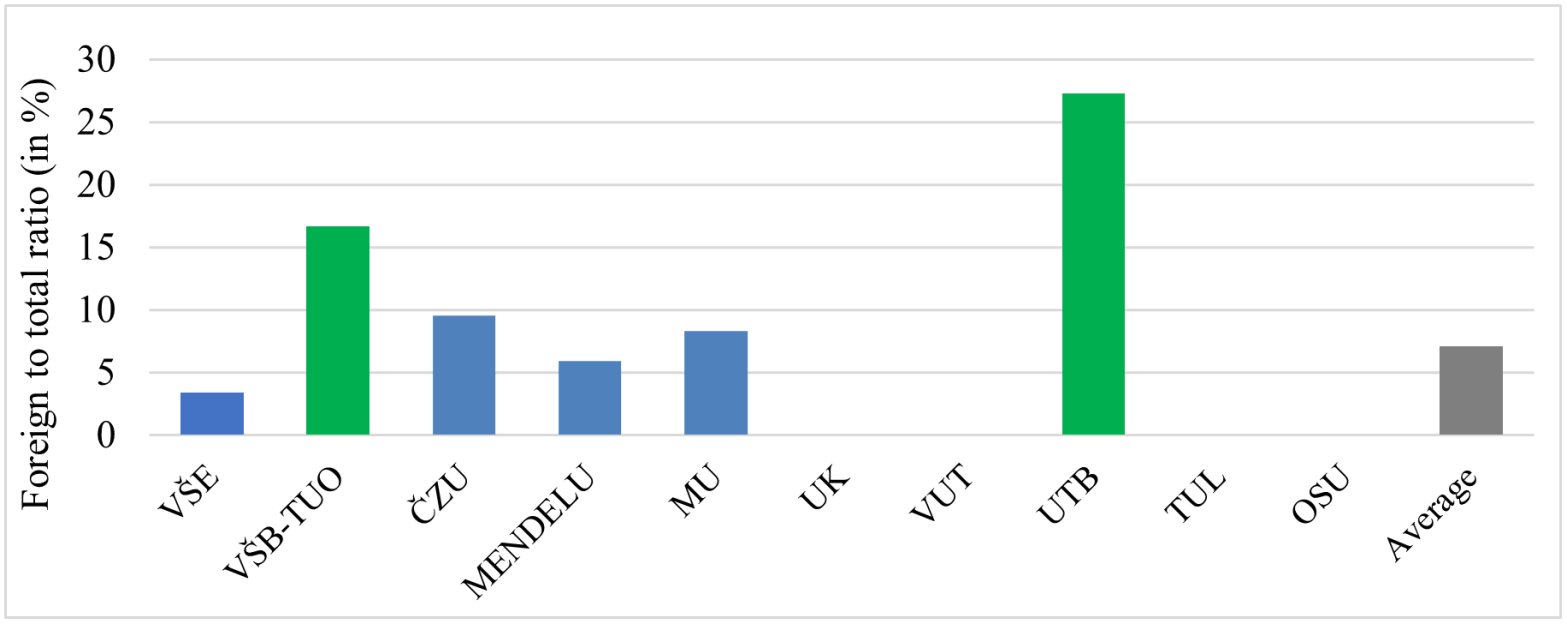
Internationalization of institutions offering appointments. *Note:* See S1 Table for the list of institutions and their abbreviations.

Most applicants came from institutions in the Czech Republic. Internationalization remains low, with only 6% of applicants coming from other countries. Almost all foreign applicants came from institutions in Slovakia, apart from a single Swede. This finding may be somewhat unexpected, given that Polish academics frequently pursue habilitation at Czech academic institutions. Fig 4 shows the share of applicants from foreign institutions in relation to the total number of appointments (the ratio of foreign applicants to total applicants). Only six out of the ten institutions offering appointments had applicants from foreign institutions, mostly from Slovakia. Thus, the ability of Czech institutions to attract foreign applicants remains low. This is similar to the situation in Germany, where international professors account for only 7.2% of university professors [127]. The most open institutions in relative terms are UTB with 27.3% of foreign applicants and VŠB-TUO with 16.7% of foreign applicants. Therefore, lagging Czech institutions should increase their internationalization efforts in the future.

**Fig 5:**
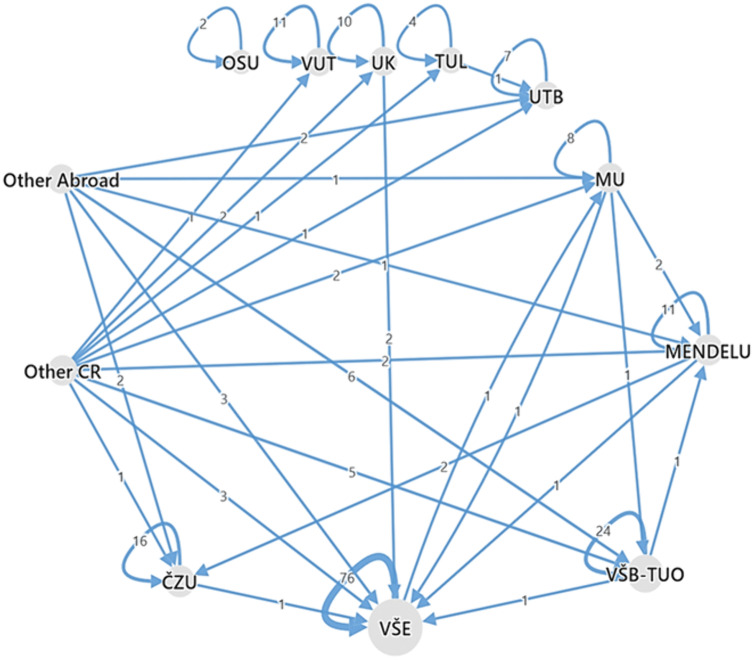
Migration of applicants across institutions. *Note:* See S1 Table for the list of institutions and their abbreviations.

The migration map in Fig 5 confirms that applicants tend to apply for professorships at their home institutions (‘home bias’), and that the flow across institutions remains very low. In many cases, only one applicant migrated between institutions during the 22-year period covered by our data. In four cases (VŠE, VŠB-TUO, ČZU, and MENDELU) more than one applicant from the same sending institution migrated to a receiving institution. Moreover, some institutions are isolated in this sense, such as OSU, VUT, TUL, and, rather surprisingly, Charles University in Prague (UK), which is the highest ranked university in the Czech Republic and one of the oldest universities in Europe. More details on migration flows across the institutions can be found in S2 Table.

**Fig 6:**
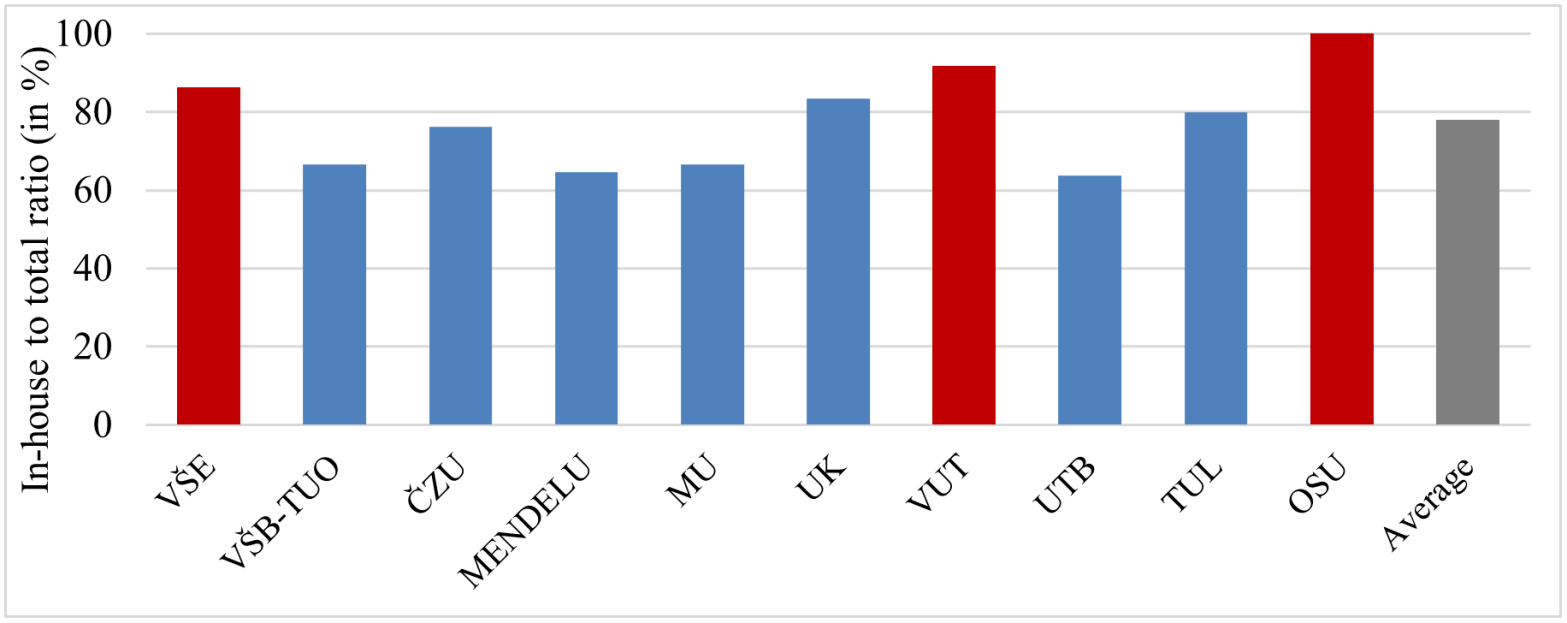
Proportion of in-house applicants. *Note:* See S1 Table for a list of institutions and their abbreviations. The institutions with the highest proportion of internal applicants are highlighted in red.

Fig 6 shows the proportion of in-house applicants. The ratio of in-house applicants to the total number of applicants, which measures the proportion of applicants from their home universities, suggests that some universities were less open to applicants from other universities and mostly promote internal candidates. The most prominent examples are OSU (100%), VUT (92%), and the largest provider of appointments, VŠE (86%), which is a dominant player in the market. In contrast, the universities that were most open to external applicants were UTB (64%), MENDELU (65%), MU (67%), and VŠB-TUO (67%), which was the second largest provider of appointments (Fig 6).

In principle, the identified ‘home bias’ could be prevalent for several reasons, including (i) search costs, as applicants must search for information about formal requirements at other institutions; (ii) information asymmetry, as internal applicants may receive internal information from academic board members and previous applicants at home institutions; (iii) fear of the unknown, as applicants often know at least some of the academic board members at home institutions, whereas they have limited information about procedures and members at other institutions; and (iv) pressure from university/faculty leadership to seek appointments at home institutions. Unfortunately, the available data do not allow us to determine which of these and other possible reasons for preferring a home institution was the most important for applicants. However, we have found that for internal applicants, the relative success rate was 78.7% of all appointment procedures in which they participated, while for external applicants it was 70.2%.

### 4.3. Publishing performance analysis using micro data

**Fig 7:**
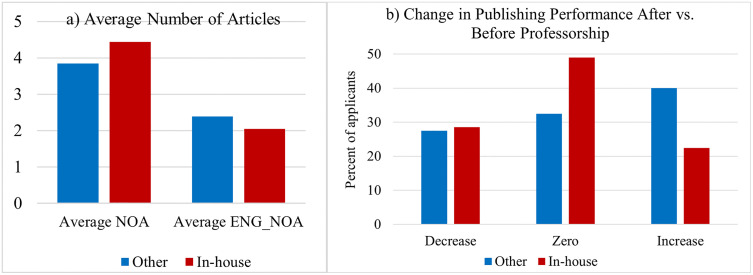
Publishing performance: The role of the home institution.

Naturally, the question arises: Is there a difference in publishing performance between internal and external applicants? To answer this question, we used a micro-level database. We examined the publishing performance of individual applicants in the WoS database, focusing on two performance indicators. First, the total number of articles was weighted by the number of authors (NOA). Second, the number of articles written in English was weighted by the number of authors (ENG_NOA). Fig 7(a) summarizes the results of this analysis. We found that being an internal applicant had little effect on publication performance. On average, internal applicants had more publications but were less internationalized in terms of their research output, as they produced fewer publications in English. This suggests that internal applicants may have focused more on local journals than on international ones, which could decrease the internationalization of research produced at Czech institutions. However, since our analysis primarily relies on publication counts and language as proxies for performance, conclusions about research quality and international competitiveness should be interpreted with some caution.

To determine the effect of the application procedure on publishing performance, we calculated the difference between the average number of articles published three years after the procedure and the average number of articles published three years before the procedure. A decrease in this indicator may signal lower motivation to publish, whereas an increase may signal that the procedure has boosted one’s research career. Our results suggest that the effect is different for in-house applicants than for other (i.e., migrating) applicants. Based on Fig 7(b), it seems that in-house applicants have a greater tendency to stagnate or even decline in terms of publication performance than the others. For them, this procedure may be a way to strengthen their position at their home institution. For applicants from other institutions, on the contrary, the procedure acts rather as a career accelerator.

The following analyses focused on the distribution of applications over time and across institutions to identify which institutions were driving this change. First, we discuss the number of appointment procedures across institutions over time with a frequency of one year (see S3 Table for details). We use the year in which the appointment process began. The highest number of procedures (21) began in 2004 and 2009, and the highest number of procedures at individual institutions (11) occurred at the VŠE in 2004. The first appointment procedure at UTB was conducted in 2010 and since then this university has remained active in this area. It is notable that the last appointment procedure at OSU occurred in 2004 and at TUL in 2012. Since 2012, only eight institutions in the Czech Republic have been involved in appointment procedures for economics professors.

To assess the publication effort of applicants over time, we calculated the total number of articles weighted by the number of authors at a given institution for each year of the sample. The results are presented in S4 Table. They allow us to identify the effects of the reform of the methodology for evaluating R&D outcomes in the Czech Republic. This reform changed the calculation of the value of articles published in WoS journals in 2009. The continuous pressure on universities from the Czech Ministry of Education, Youth and Sports seems to have produced results since 2012, as the average number of articles has been growing since then (see S4 Table for details).

**Fig 8:**
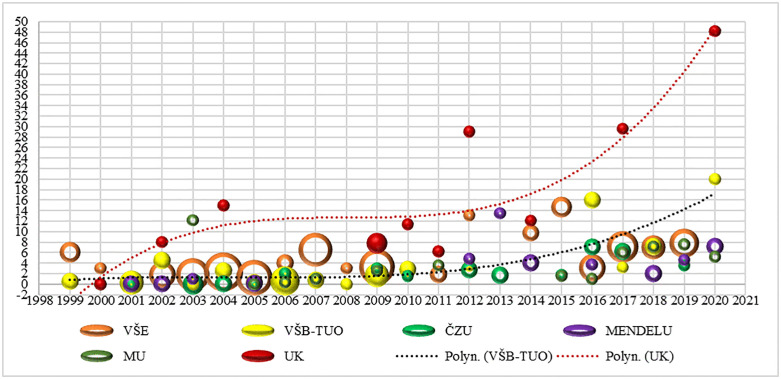
Average number of articles by year at the top six institutions.

Finally, Fig 8 illustrates the publication performance measured by the average number of articles at an institution with respect to the number of applicants at the HEI level. We present the results for the top six institutions in terms of number of applicants over the 22-year period from 1999 to 2020. To demonstrate the representativeness of the averages, the size of the bubbles reflects the number of applicants in a given year. Therefore, the bubble size is larger when the number of applicants is higher. In this way, we can determine the effect of the reforms on the methodology of evaluating R&D results in the Czech Republic. The largest increase in the trend after the reforms occurred at Charles University, followed by VŠB-TUO. The developments at these two institutions are highlighted in the graph with third-order polynomial trends.

#### Pairwise correlation analysis.

To further examine the relationships between publication performance and its potential determinants, as identified in our data set, we proceed with correlation and regression analyses. When calculating pairwise correlations, we find a significant positive correlation between the total number of articles (NOA_T) and the number of articles written in English (ENG_NOA_T). A negative correlation between the total number of articles and sex (SEX) at the 5% significance level suggests that women seek professorship with fewer publications, but this is not the case for articles in English. A negative correlation between sex and being an internal applicant (IN) at the 10% significance level suggests that women are less likely to seek a professorship at their home university and prefer to migrate more often. Our data shows that 30% of women migrated to other institutions during the examined period, while only 19% of men did. Finally, we find significant correlations between the year of the professorship attempt (Year) and both the total number of publications and the number of publications in English at the 1% significance level. The findings indicate that the implementation of enhanced standards through reform initiatives has resulted in an improvement in the publishing performance of leading academic professionals (Table 1).

**Table 1:** Correlation analysis.

	NOA_T	ENG_NOA_T	IN	SEX	Year
NOA_T	1				
ENG_NOA_T	0.7832*** (0.0000)	1			
IN	0.0369 (0.5891)	−0.0287 (0.6746)	1		
SEX	−0.1480** (0.0296)	−0.0805 (0.2388)	−0.1233** (0.0706)	1	
Year	0.4031*** (0.0000)	0.4653*** (0.0000)	−0.0617 (0.3667)	−0.0324 (0.6357)	1

p-values in parentheses, significance levels: *** = 1%; ** = 5%; * = 10%

#### Regression analysis.

Our estimation results for the baseline model, presented in Table 2 as Model 1, suggest that the number of published articles increased by 0.43 per year on average at the 1% significance level, indicating an increasing publication performance of applicants. This finding aligns with previous studies on the Czech Republic [104,106]. The year effects also remain a significant determinant for the number of foreign language publications, with an increase of 0.37 per year at the 1% significance level (Table 3 Model 4), suggesting an increasing tendency to publish in English over time. Nevertheless, this positive tendency does not necessarily imply an improvement in research quality or genuine international competitiveness because our investigation does not consider citation metrics (see [106] for the discussion of the problem with research quality in the Czech Republic). In general, women entered the professorship procedure with a lower number of publications than men. This effect is significant at the 5% level. However, we do not find this effect to be statistically significant for English publications. This may indicate that women are less prone to publishing in local journals than men in our sample. Since we do not find a significant difference between the promotion success of women and men (the corresponding relative success rates were 76.8% and 76.9%), our finding does not support the female devaluation theory. This finding is consistent with a recent study in psychology by Lutter et al. [128], who found that ‘women tend to benefit more from their scholarly publications than men’ based on an analysis of data on professors in Germany. This finding also suggests that there may not be a gender bias in favor of men, even though they represent a significant majority on scientific boards. Conversely, if we consider research productivity, measured by the number of peer-reviewed articles, as a key criterion for obtaining a professorship, we can expect a positive gender premium for women, which would be consistent with the findings of Card et al. [129]. In principle, our results would also be consistent with some findings in economics that lower research productivity of women is associated with greater recognition [130,131] or quality of their publications [132], although we do not measure the number of citations or the readability of articles. Our findings indicate that while internal candidates may produce a greater overall number of articles, which are less likely to be in English, this effect is not statistically significant in our models.

**Table 2:** Determinants of overall publishing performance (NOA_T).

VARIABLES	Model1	Model2	Model3
IN	0.737	0.369	0.576
	(1.009)	(1.012)	(0.922)
SEX	−1.960**	−2.005**	−1.454*
	(0.948)	(0.938)	(0.871)
Year	0.433***	0.453***	0.430***
	(0.067)	(0.068)	(0.062)
D_Capital		2.538**	
		(0.980)	
D_Structural		1.240	
		(1.287)	
D_UK			10.922***
			(1.653)
D_MU			−1.115
			(1.662)
Constant	−866.162***	−907.063***	−859.608***
	(134.947)	(137.019)	(123.866)
Observations	216	216	216
R-squared	0.183	0.209	0.327

Standard errors in parentheses

*** p < 0.01, ** p < 0.05, * p < 0.1

**Table 3:** Determinants of research visibility and impact (ENG_NOA_T).

VARIABLES	Model4	Model5	Model6
IN	−0.100	−0.236	−0.234
	(0.729)	(0.740)	(0.642)
SEX	−0.746	−0.762	−0.323
	(0.685)	(0.686)	(0.606)
Year	0.369***	0.376***	0.366***
	(0.048)	(0.050)	(0.043)
D_Capital		0.916	
		(0.717)	
D_Structural		0.418	
		(0.941)	
D_UK			9.119***
			(1.151)
D_MU			−0.931
			(1.157)
Constant	−739.297***	−753.428***	−733.830***
	(97.493)	(100.204)	(86.218)
Observations	216	216	216
R-squared	0.221	0.227	0.404

Standard errors in parentheses

*** p < 0.01, ** p < 0.05, * p < 0.1

Next, we perform our first robustness test related to the structural characteristics of the regions where the procedure took place. The estimation results presented in Table 2 Model 2 and Table 3 Model 5 show robust effects of the baseline model variables. In addition, we identify a significant effect of capital at the 5% significance level, which means that applicants to universities located in Prague tend to enter the professorship with a greater number of publications that applicants in other regions. This result is consistent with our expectations based on the core-periphery theory. As the capital city, Prague is home to most government institutions, including the Ministries of Finance, Industry and Trade, Labor and Social Affairs, the Central Bank, and the economic research institutes of the Academy of Sciences. These institutions provide greater opportunities for economic research cooperation and better chances of obtaining grants. Nevertheless, we do not find any statistically significant effect for the structurally affected Moravian-Silesian Region, which suggests that the number of articles among applicants in this region does not differ from the other regions and the Moravian-Silesian Region does not suffer from its structural characteristics in terms of applicant publication quantity.

In addition, the results of the second robustness test (Table 2 Model 3 and Table 3 Model 6) confirm the robustness of the baseline model results and provide further details related to the first robustness test. We find a significant positive effect of D_UK at the 1% significance level, suggesting that applicants at Charles University in Prague enter the procedure with a higher number of publications compared to applicants from other universities. This holds for both the total number of published articles, as well as those published in English, which is consistent with the prestige of this institution. Surprisingly, we do not find a similar effect for the second A-ranked university in our sample, as the coefficient for Masaryk University in Brno is statistically insignificant. Overall, it seems that the A-ranking of the university is not a sufficient condition for a higher number of publications among applicants. This may reflect the fact that Charles University is considered a highly selective, nationally leading (“flagship”) university. Founded in 1348, it is one of the world’s oldest continuously operating universities. It is consistently ranked among the top Central and Eastern European universities in international rankings, particularly in economic sciences. Its joint workplace with the Economics Institute of the Czech Academy of Sciences, CERGE-EI (Center for Economic Research and Graduate Education—Economics Institute), employs research standards similar to those of top U.S. or Western European universities.

## 5. Conclusion

In principle, promotion to full professor can be based on two different career models: the competence model and the competition model. Under the competence model, all qualified candidates can be promoted to full professor based on an evaluation of their academic performance. In contrast, the competition model requires candidates to compete for a limited number of vacancies. Both career models have their advantages and disadvantages [133]. The competence model is considered fairer and more attractive to promising young academics and for women candidates. However, if candidates can seek promotion at their own university, it may lead to less mobility between universities. Additionally, the competence model reduces HEIs’ control over the specific research and teaching specialties of full professors, which can lead to a mismatch between the institution’s needs and its staff’s academic focus. The competence model is used in the Czech Republic. However, unlike in Poland, evaluations are performed at the university level, and professorship appointments are strictly regulated by national accreditation of institutions’ appointment rights. Furthermore, the (full) Professor degree is nationally recognized, regardless of which domestic university awarded it.

We found that the Czech competence promotion system leads to low domestic academic mobility as candidates in such a system are less likely to seek a professorship at another university. This is consistent with the findings for Norway [133]. Additionally, our research revealed no significant differences in the research productivity or visibility of internal and external candidates for a professorship. The same is true for their performance after being appointed. These results are surprising given the existing literature on in-group bias and favoritism in academic promotions. We believe that this can be explained by the fact that, in the Czech Republic, university scientific councils include both internal and external members, who may engage in the exchange of favors between universities [134]. For example, the Scientific Council of the VSB – Technical University of Ostrava had 15 external members out of 43 total members in 2025. Thirteen of these members were from other public universities, including three rectors and three vice rectors. Similarly, 22 of the 62 members of the Masaryk University Scientific Council were from other public universities, including five rectors and four vice-rectors. When there is considerable overlap in the membership of Czech universities’ scientific councils [135], the members who decide on granting professorships may have reciprocal expectations.

Although the mobility of candidates for professorships does not seem to impact the productivity or visibility of their research, we have documented several weak points in Czech academia. Research productivity and visibility remain low, though the national performance-based funding model has helped improve the situation when implemented in universities. Furthermore, the promotion system does not attract foreign applicants, and women are underrepresented among candidates for professorships.

Some of these issues were discussed extensively over a decade ago [136,137]. When the amendment to the Czech Higher Education Act was being prepared in 2013, arguments were made that the national system of professorships was unattractive to foreign and domestic applicants, motivated professors to hold academic second jobs, prevented universities from opening new programs according to their needs, encouraged clientelism, and could have negative effects on less developed regions of the country. These factors hindered knowledge production and dissemination. Despite past reform efforts, however, there have been no fundamental changes to the system [138], as universities rejected the proposal to abolish lifetime professorships and replace the existing competence promotion model with the competition model.

We agree that significantly altering the current promotion model could cause major disruption in Czech academia, already plagued by underfunding as it is, resulting in negative consequences such as a loss of motivation among junior academics pursuing academic careers and an increased risk of a gender promotion gap. However, we believe several important measures can be taken within the existing system to improve the research performance and impact of future university professors. Scientific councils should be composed primarily of external members from the international community rather than representatives from other domestic universities. For example, the Scientific Council of Charles University had 24 external members in 2025, but the only foreigners were four members from Slovak universities and one professor born in Germany who worked at a Czech research institute at the time. Although Czech universities have recently established international scientific councils, these councils only act as independent advisory bodies to the university leadership. To support the internationalization of candidates, international collaborative networks, publications with international co-authors and postdoctoral mobility should carry more weight in the evaluation criteria. However, mandatory long-term mobility or postdoctoral positions abroad may increase the “mobility gap” between women and men [139].

The problem of a small number of foreigners and experienced local practitioners working at universities is exacerbated by the demanding habilitation process required to secure a senior academic position with competitive compensation [140]. In 2016, therefore, selected public universities were granted the right to establish the position of extraordinary professor. This non-degree academic position was reintroduced to foster the recruitment of domestic and foreign experts for long-term academic positions. However, its use in academia remains rare due to administrative requirements and financial demands, resulting in low interest from HEIs [141]. For example, 13 universities currently have the right to establish the position of extraordinary professor of economics, yet we are not aware of any appointments. Although Charles University had 798 full professors in 2023, it employed only nine extraordinary professors. To increase the use of extraordinary professors, all HEIs with accredited degree programs should be allowed to establish the position. However, this would require deregulation and a change in the law. It would significantly alter the current competence promotion model by adding elements of a prospective competition model, as is currently the case in Slovakia.

Finally, spatial disparities and inequalities in the academic market can negatively impact regional growth and development because knowledge diffusion is important for economic and societal development [142–144]. If promotion rights are limited to a few selected universities in traditional academic centers, it can have adverse effects on innovation, entrepreneurship, employment, and growth in more peripheral areas [145–148]. Therefore, we propose reducing the spatial concentration of HEIs authorized to grant professorships and expanding professorships by modifying the existing competence promotion model. This would reduce the demand for professors with second jobs and enable structurally affected regions to benefit from a stronger accumulation of human capital, as well as the production and dissemination of knowledge. It could also help mitigate the brain drain these regions experience. For example, despite discussions about establishing a public university in the Karlovy Vary region, it still lacks one. While 17.6% of the Czech population had a university degree in 2021, according to the latest data, only 9.6% of the population in the Karlovy Vary region did. As we have demonstrated, structurally affected regions have not been found to negatively impact the research productivity and visibility of professorship candidates. Therefore, degradation of academic standards and requirements would not necessarily be a concern. Additionally, the observed effects of capital city location and institutional prestige suggest creating or enhancing opportunities for collaboration with government institutions and research-oriented institutes to support academic research performance. According to the literature on public sector relocation and fiscal decentralization [149–151], moving central government functions from one part of the country to another could allow universities on the periphery to interact more deeply with the relocated institutions and gain better access to research funds. Currently, nearly all government institutions, including the Czech Science Foundation and the Technology Agency of the Czech Republic are based in the capital city (Prague). This puts universities and researchers in other regions at a significant disadvantage. Therefore, public sector relocation could improve the situation.

Nevertheless, our conclusions about research quality and international competitiveness are based solely on publication counts and language as proxies for performance. For instance, one could argue that, on average, internal candidates publish fewer articles in foreign languages, but in journals with higher Journal Impact Factors or Article Influence Scores than external candidates. After becoming professors, these candidates may also be more likely to reduce their publication output while publishing in higher-quality or more impactful journals. Therefore, future research should incorporate citation metrics and journal quality into the analysis to mitigate the risk of overstating findings.

## Supporting information

S1 Table Institutions and acronyms. (DOCX)

S2 Table Migration of applicants across institutions. (DOCX)

S3 Table Appointment procedures at institutions over time. (DOCX)

S4 Table Publishing performance of applicants at universities over time. (DOCX)

S5 Dataset Dataset. (XLSX)
