# Supplementary material for: To be a professor: Academic mobility and publishing performance
Source: PLoS One. 2025 Nov 17;20(11):e0336133. doi: 10.1371/journal.pone.0336133 (PMC12622835; doi:10.1371/journal.pone.0336133)
Supplement: S1 Table — (DOCX) [file pone.0336133.s001.docx]

**S1 Table. Institutions and acronyms**

| Acronym | Name of the institution | |
| --- | --- | --- |
|  | Czech | English |
| VŠE | Vysoká škola ekonomická v Praze | Prague University of Economics and Business |
| VŠB-TUO | Vysoká škola báňská – Technická univerzita Ostrava | VSB – Technical University of Ostrava |
| ČZU | Česká zemědělská univerzita v Praze | Czech University of Life Sciences, Prague |
| MENDELU | Mendelova univerzita v Brně | Mendel University in Brno |
| MU | Masarykova univerzita | Masaryk University |
| UK | Univerzita Karlova | Charles University |
| VUT | Vysoké učení technické v Brně | Brno University of Technology |
| UTB | Univerzita Tomáše Bati ve Zlíně | Tomas Bata University in Zlín |
| TUL | Technická univerzita v Liberci | Technical University of Liberec |
| *TUK* | Technická univerzita v Košiciach | Technical University of Košice |
| *UAD Trenčín* | Trenčianská univerzita Alexandra Dubčeka v Trenčíně | Alexander Dubček University of Trenčín |
| *EUBA* | Ekonomická univerzita v Bratislave | University of Economics in Bratislava |
| SUO | Slezská univerzita v Opavě | Silesian University in Opava |
| OSU | Ostravská univerzita | University of Ostrava |
| *UMB* | Univerzita Mateje Bela v Banskej Bystrici | Matej Bel University, Bánská Bystrica |
| UPCE | Univerzita Pardubice | University of Pardubice |
| *SPU Nitra* | Slovenská poľnohospodárska univerzita v Nitre | Slovak University of Agriculture in Nitra |
| UHK | Univerzita Hradec Králové | University of Hradec Králové |
| ČNB | Česká národní banka | Czech National Bank |
| VŠFS | Vysoká škola finanční a správní | University of Finance and Administration |
| VŠTE | Vysoká škola technická a ekonomická v Českých Budějovicích | Institute of Technology and Business in České Budějovice |
| *PU Prešov* | Prešovská univerzita v Prešove | University of Presov |
| *SAV Bratislava* | Slovenská akadémia vied, Ekonomický ústav | Slovak Academy of Sciences, Institute of Economic Research |
| VŠEM | Vysoká škola ekonomie a managementu | University of Economics and Management, Prague |
| ZČU | Západočeská univerzita v Plzni | University of West Bohemia |
| JČU | Jihočeská univerzita v Českých Budějovicích | University of South Bohemia in České Budějovice |
| VŠCHT | Vysoká škola chemicko-technologická v Praze | University of Chemistry and Technology, Prague |
| NM Praha | Národní muzeum | National Museum |

*Note:* Acronyms for foreign institutions are in italics.
