# Supplementary material for: To be a professor: Academic mobility and publishing performance
Source: PLoS One. 2025 Nov 17;20(11):e0336133. doi: 10.1371/journal.pone.0336133 (PMC12622835; doi:10.1371/journal.pone.0336133)
Supplement: S2 Table — (DOCX) [file pone.0336133.s002.docx]

**S2 Table. Migration of applicants across institutions**

| From / To | VŠE | VŠB-TUO | ČZU | MENDELU | MU | UK | VUT | UTB | TUL | OSU | Total |
| --- | --- | --- | --- | --- | --- | --- | --- | --- | --- | --- | --- |
| VŠE | 76 |  |  |  | 1 |  |  |  |  |  | 77 |
| VŠB-TUO | 1 | 24 |  | 1 |  |  |  |  |  |  | 26 |
| ČZU | 1 |  | 16 |  |  |  |  |  |  |  | 17 |
| MENDELU | 1 |  | 2 | 11 |  |  |  |  |  |  | 14 |
| MU | 1 | 1 |  | 2 | 8 |  |  |  |  |  | 12 |
| UK | 2 |  |  |  |  | 10 |  |  |  |  | 12 |
| VUT |  |  |  |  |  |  | 11 |  |  |  | 11 |
| UTB |  |  |  |  |  |  |  | 7 |  |  | 7 |
| TUL |  |  |  |  |  |  |  | 1 | 4 |  | 5 |
| *TUK* |  | 2 |  |  |  |  |  | 1 |  |  | 3 |
| *UAD Trenčín* |  | 1 | 1 |  |  |  |  | 1 |  |  | 3 |
| *EUBA* | 1 | 1 |  |  | 1 |  |  |  |  |  | 3 |
| SUO |  | 2 |  | 1 |  |  |  |  |  |  | 3 |
| OSU |  |  |  |  |  |  |  |  |  | 2 | 2 |
| *UMB* | 1 | 1 |  |  |  |  |  |  |  |  | 2 |
| UPCE |  |  |  | 1 | 1 |  |  |  |  |  | 2 |
| *SPU Nitra* |  |  | 1 | 1 |  |  |  |  |  |  | 2 |
| UHK | 1 | 1 |  |  |  |  |  |  |  |  | 2 |
| ČNB | 1 |  |  |  |  | 1 |  |  |  |  | 2 |
| VŠFS | 1 | 1 |  |  |  |  |  |  |  |  | 2 |
| VŠTE |  |  |  |  |  |  | 1 |  |  |  | 1 |
| *PU Prešov* |  | 1 |  |  |  |  |  |  |  |  | 1 |
| *SAV Bratislava* |  |  |  |  |  |  |  | 1 |  |  | 1 |
| *KVUT Stockholm* | 1 |  |  |  |  |  |  |  |  |  | 1 |
| VŠEM |  |  |  |  | 1 |  |  |  |  |  | 1 |
| ZČU |  |  |  |  |  |  |  |  | 1 |  | 1 |
| JČU |  |  | 1 |  |  |  |  |  |  |  | 1 |
| VŠCHT |  | 1 |  |  |  |  |  |  |  |  | 1 |
| NM Praha |  |  |  |  |  | 1 |  |  |  |  | 1 |
| Total | 88 | 36 | 21 | 17 | 12 | 12 | 12 | 11 | 5 | 2 | 216 |

Note: Colours in the table range from red (lowest) to green (highest).
