# Supplementary material for: To be a professor: Academic mobility and publishing performance
Source: PLoS One. 2025 Nov 17;20(11):e0336133. doi: 10.1371/journal.pone.0336133 (PMC12622835; doi:10.1371/journal.pone.0336133)
Supplement: S3 Table — (DOCX) [file pone.0336133.s003.docx]

**S3 Table. Appointment procedures at institutions over time**

|  | | | | | | | | | | | | | | | | | | | | | | |
| --- | --- | --- | --- | --- | --- | --- | --- | --- | --- | --- | --- | --- | --- | --- | --- | --- | --- | --- | --- | --- | --- | --- |
| Institution / Year | 1999 | 2000 | 2001 | 2002 | 2003 | 2004 | 2005 | 2006 | 2007 | 2008 | 2009 | 2010 | 2011 | 2012 | 2013 | 2014 | 2015 | 2016 | 2017 | 2018 | 2019 | 2020 |
| VŠE | 3 | 1 | 1 | 5 | 8 | 11 | 9 | 2 | 8 | 1 | 9 |  | 2 | 1 |  | 2 | 3 | 5 | 7 | 4 | 6 |  |
| VŠB-TUO | 2 |  | 4 | 2 | 2 | 2 | 2 | 6 | 2 | 1 | 4 | 2 |  | 1 |  |  |  | 2 | 1 | 2 |  | 1 |
| ČZU |  | 1 | 1 |  | 3 | 2 | 1 | 1 |  |  | 1 | 1 |  | 2 | 2 |  | 1 | 2 | 2 |  | 1 |  |
| MENDELU |  |  | 2 | 2 | 1 |  | 2 |  |  |  |  |  |  | 1 | 1 | 2 |  | 1 |  | 2 | 1 | 2 |
| MU |  |  |  |  | 1 |  | 1 | 1 | 1 |  | 1 |  | 1 |  |  |  | 1 | 1 | 1 | 1 | 1 | 1 |
| UK |  | 1 |  | 1 |  | 1 |  |  |  |  | 3 | 1 | 1 | 1 |  | 1 |  |  | 1 |  |  | 1 |
| VUT |  | 1 |  | 1 |  | 3 |  |  |  |  | 1 | 2 |  | 1 |  |  |  | 1 |  | 1 |  | 1 |
| UTB |  |  |  |  |  |  |  |  |  |  |  | 3 | 1 |  |  |  | 2 | 1 |  | 1 | 3 |  |
| TUL |  |  |  | 1 |  | 1 |  |  |  |  | 2 |  |  | 1 |  |  |  |  |  |  |  |  |
| OSU |  | 1 |  |  |  | 1 |  |  |  |  |  |  |  |  |  |  |  |  |  |  |  |  |
| Total | 5 | 5 | 8 | 12 | 15 | 21 | 15 | 10 | 11 | 2 | 21 | 9 | 5 | 8 | 3 | 5 | 7 | 13 | 12 | 11 | 12 | 6 |

Note: Colours in the table range from red (lowest) to green (highest).
