# Supplementary material for: To be a professor: Academic mobility and publishing performance
Source: PLoS One. 2025 Nov 17;20(11):e0336133. doi: 10.1371/journal.pone.0336133 (PMC12622835; doi:10.1371/journal.pone.0336133)
Supplement: S4 Table — (DOCX) [file pone.0336133.s004.docx]

| **S4 Table. Publishing performance of applicants at universities over time** | | | | | | | | | | | | | | | | | | | | | | |
| --- | --- | --- | --- | --- | --- | --- | --- | --- | --- | --- | --- | --- | --- | --- | --- | --- | --- | --- | --- | --- | --- | --- |
| Institution / Year | 1999 | 2000 | 2001 | 2002 | 2003 | 2004 | 2005 | 2006 | 2007 | 2008 | 2009 | 2010 | 2011 | 2012 | 2013 | 2014 | 2015 | 2016 | 2017 | 2018 | 2019 | 2020 |
| VŠE | 6.1 | 3.0 | 0.0 | 1.7 | 1.7 | 2.3 | 1.3 | 4.0 | 6.5 | 3.0 | 3.2 |  | 1.9 | 13.1 |  | 9.8 | 14.7 | 3.1 | 7.1 | 6.9 | 7.7 |  |
| VŠB-TUO | 0.5 |  | 0.3 | 4.5 | 0.0 | 2.5 | 0.3 | 0.6 | 0.8 | 0.0 | 1.7 | 2.8 |  | 3.5 |  |  |  | 16.0 | 3.2 | 7.2 |  | 20.0 |
| ČZU |  | 0.0 | 0.0 |  | 0.0 | 0.0 | 0.0 | 2.0 |  |  | 3.0 | 1.5 |  | 2.7 | 1.7 |  | 1.8 | 7.1 | 6.2 |  | 3.6 |  |
| MENDELU |  |  | 0.0 | 0.0 | 1.0 |  | 0.1 |  |  |  |  |  |  | 4.8 | 13.5 | 4.0 |  | 3.7 |  | 2.0 | 4.6 | 7.1 |
| MU |  |  |  |  | 12.2 |  | 0.0 | 0.3 | 1.0 |  | 2.5 |  | 3.6 |  |  |  | 1.5 | 1.0 | 6.0 | 7.1 | 7.6 | 5.2 |
| UK |  | 0.0 |  | 8.0 |  | 15.0 |  |  |  |  | 7.7 | 11.3 | 6.2 | 29.0 |  | 12.1 |  |  | 29.6 |  |  | 48.2 |
| VUT |  | 0.0 |  | 0.0 |  |  |  |  |  |  | 0.0 | 0.1 |  | 1.3 |  |  |  | 2.4 |  | 13.9 |  | 6.9 |
| UTB |  |  |  |  |  |  |  |  |  |  |  | 0.8 | 0.0 |  |  |  | 2.7 | 0.0 |  | 6.3 | 13.9 |  |
| TUL |  |  |  | 0.0 |  | 17.0 |  |  |  |  | 0.5 |  |  | 1.5 |  |  |  |  |  |  |  |  |
| OSU |  | 0.0 |  |  |  | 0.0 |  |  |  |  |  |  |  |  |  |  |  |  |  |  |  |  |

Note: Colours in the table range from red (lowest) to green (highest)
